# *Drosophila melanogaster* Activating Transcription Factor 4 Regulates Glycolysis During Endoplasmic Reticulum Stress

**DOI:** 10.1534/g3.115.017269

**Published:** 2015-02-13

**Authors:** Ji Eun Lee, McKenna Oney, Kimberly Frizzell, Nitin Phadnis, Julie Hollien

**Affiliations:** Department of Biology, University of Utah, Salt Lake City, Utah 84112

**Keywords:** unfolded protein response, endoplasmic reticulum stress, Atf4, glycolysis, metabolism

## Abstract

Endoplasmic reticulum (ER) stress results from an imbalance between the load of proteins entering the secretory pathway and the ability of the ER to fold and process them. The response to ER stress is mediated by a collection of signaling pathways termed the unfolded protein response, which plays important roles in development and disease. Here we show that in *Drosophila melanogaster* S2 cells, ER stress induces a coordinated change in the expression of genes involved in carbon metabolism. Genes encoding enzymes that carry out glycolysis were up-regulated, whereas genes encoding proteins in the tricarboxylic acid cycle and respiratory chain complexes were down-regulated. The unfolded protein response transcription factor Atf4 was necessary for the up-regulation of glycolytic enzymes and *Lactate dehydrogenase (Ldh)*. Furthermore, Atf4 binding motifs in promoters for these genes could partially account for their regulation during ER stress. Finally, flies up-regulated *Ldh* and produced more lactate when subjected to ER stress. Together, these results suggest that Atf4 mediates a shift from a metabolism based on oxidative phosphorylation to one more heavily reliant on glycolysis, reminiscent of aerobic glycolysis or the Warburg effect observed in cancer and other proliferative cells.

The endoplasmic reticulum (ER) is responsible for folding and processing proteins entering the secretory pathway. Because the flux of proteins through the ER varies considerably among cell types and in different conditions, cells maintain a balance between the load on the ER and its protein folding capacity. However, a number of biochemical, physiological, and pathological stimuli can disrupt this balance, resulting in ER stress. To re-establish ER homeostasis, the unfolded protein response (UPR) is activated (Walter and Ron 2011; Moore and Hollien 2012). This network of pathways up-regulates genes encoding ER-specific chaperones and other proteins involved in protein secretion (Travers *et al.* 2000) while also attenuating protein translation (Shi *et al.* 1998; Harding *et al.* 1999) and degrading certain ER-associated mRNAs (Hollien and Weissman 2006; Hollien *et al.* 2009). The UPR is broadly conserved across eukaryotes (Hollien 2013) and is essential for normal development in several model organisms, particularly for professional secretory cells, where it is thought to be important for the establishment and maintenance of high levels of protein secretion (Moore and Hollien 2012). It is also induced during many metabolic conditions, including diabetes, hyperlipidemia, and inflammation, and has been implicated in various cancers, especially in the growth of large tumors that rely on an effective response to hypoxia (Wang and Kaufman 2012; 2014).

The UPR is carried out by three main signaling branches. One of these is initiated by the ER transmembrane protein inositol-requiring enzyme 1 (Ire1) (Cox *et al.* 1993; Mori *et al.* 1993). When activated by ER stress, the cytosolic endoribonuclease domain of Ire1 cleaves the mRNA encoding the transcription factor Xbp1, thereby initiating an unconventional splicing event that produces the mRNA template encoding a highly active form of Xbp1 (Yoshida *et al.* 2001; Calfon *et al.* 2002). Ire1 also cleaves other mRNAs associated with the ER membrane through a pathway that is particularly active in *Drosophila* cells and that may reduce the load on the ER (Hollien and Weissman 2006; Gaddam *et al.* 2013). A second sensor of ER stress, activating transcription factor 6, is activated by proteolysis, which releases it from the ER membrane and allows it to travel to the nucleus and regulate gene expression (Haze *et al.* 1999; Wang *et al.* 2000). Finally, protein kinase RNA−like ER kinase (Perk) phosphorylates eukaryotic initiation factor 2 alpha, leading to a general attenuation of protein synthesis as well as the translational up-regulation of certain mRNAs that contain upstream open reading frames (ORFs) in their 5′ untranslated regions (Harding *et al.* 2000). Activating transcription factor 4 (Atf4) is among those proteins that are up-regulated translationally during ER stress and regulates genes involved in protein secretion as well as amino acid import and resistance to oxidative stress (Harding *et al.* 2003).

In addition to its direct effects on the protein secretory pathway, the UPR influences several other cellular pathways, including apoptosis (Logue *et al.* 2013), inflammation (Garg *et al.* 2012), and lipid synthesis (Basseri and Austin 2012). Furthermore, the UPR (particularly the Perk/Atf4 branch) appears to have close ties to mitochondrial function. For example, knockout of Mitofusin 2, a key mitochondrial fusion protein, activates Perk, leading to enhanced reactive oxygen species (ROS) production and reduced respiration (Muñoz *et al.* 2013). Atf4 also increases expression of Parkin, which mediates degradation of damaged mitochondria, protecting cells from ER stress-induced mitochondrial damage (Bouman *et al.* 2010). Despite clear links between ER stress and mitochondria, the mechanistic relationship between the UPR and mitochondrial metabolism is not well-understood.

Here we report that the UPR in *Drosophila melanogaster* S2 cells triggers a coordinated change in the expression of genes involved in carbon metabolism. The metabolism of glucose as an energy source produces pyruvate, which can then enter the mitochondria and the tricarboxylic acid (TCA) cycle to produce reducing equivalents for oxidative phosphorylation (OXPHOS). For most cells in normal conditions, the majority of ATP is produced through OXPHOS. However, in hypoxic conditions when OXPHOS is limited, cells rely heavily on glycolysis to compensate for the decrease in ATP production and convert the excess pyruvate to lactate, which then leaves the cell (Zheng 2012). This shift from OXPHOS to glycolysis is seen in a variety of cancers even when cells have access to oxygen, an effect known as aerobic glycolysis or the Warburg effect, and is thought to be a hallmark of cancer cells (Dang 2012). Aerobic glycolysis is also becoming increasingly recognized as a metabolic signature of other cell types as well, including stem cells and activated immune cells (Fox *et al.* 2005; Rafalski *et al.* 2012).

The *D. melanogaster* estrogen-related receptor is the only transcription factor known to regulate glycolytic genes in flies (Li *et al.* 2013). Its activity is temporally regulated during mid-embryogenesis to support aerobic glycolysis during larval growth (Tennessen *et al.* 2011). Moreover, a recent study found that glycolytic gene expression under hypoxic conditions in larvae is partially dependent on *D. melanogaster* estrogen-related receptor (Li *et al.* 2013). Here, we show that the UPR transcription factor Atf4 also regulates glycolytic genes, contributing to a broad regulation of metabolic gene expression during ER stress that is reminiscent of the Warburg effect.

## Materials and Methods

### Cell culture, ER stress induction, RNA interference (RNAi)

We grew *D. melanogaster* S2 cells (Invitrogen) at room temperature in Schneider’s *Drosophila* media (Invitrogen) supplemented with 10% heat-inactivated fetal bovine serum and antibiotics. To induce ER stress, we treated cells with dithiothreitol (DTT, 2 mM, 5 hr) or tunicamycin (Tm, 5 μg/mL, 16 hr) unless otherwise stated.

To deplete cells of Atf4 by RNAi, we amplified a 527-nucleotide region from the coding sequence of Atf4 (also known as cryptocephal/crc, CG8669) using primers with T7 RNA polymerase sites at the 5′ ends. This amplicon has no predicted off-target 21 nt siRNA sequences, as determined using the *Drosophila* RNAi Screening Center (http://www.flyrnai.org). We used this polymerase chain reaction (PCR) product to generate double-stranded RNA (dsRNA) by *in vitro* transcription (Megascript T7 kit; Ambion). We then incubated S2 cells with 15 μg of dsRNA in serum-free media for 45 min, replaced the serum, and allowed the cells to recover for 5 d. We retreated cells with 45 μg of dsRNA and induced ER stress 1 d after the second dsRNA treatment.

### RNA preparation and quantitative real-time PCR (qPCR)

We extracted total RNA from cells or decapitated flies using Trizol reagent (Invitrogen). For transfected cells, we subsequently subjected purified RNA to RQ1 RNase-free DNase I (Promega) treatment to remove residual plasmid DNA. We synthesized cDNA from 2 μg of total RNA, using a T_18_ primer and M-MuLV reverse transcriptase (NEB). We then performed qPCR using a Mastercycler ep realplex (Eppendorf) with SYBR Green as the fluorescent dye. We measured each sample in triplicate and normalized relative RNA levels to those of the housekeeping gene *Ribosomal protein L19 (RpL19)*. Sequences of all qPCR primers are listed in Table 1.

**Table 1:** Primers used for qPCR

Gene Name	Primer1	Primer2
RpL19	AGGTCGGACTGCTTAGTGACC	CGCAAGCTTATCAAGGATGG
Pfk	CTGCAGCAGGATGTCTACCA	GTCGATGTTCGCCTTGATCT
Tpi	GACTGGAAGAACGTGGTGGT	CGTTGATGATGTCCACGAAC
Ldh	GTGTGACATCCGTGGTCAAG	CTACGATCCGTGGCATCTTT
CG7430	TTTCGGCAGTAGCCAAGACT	ACCGTCTTCATGCCCATCT
l(1)G0255	CTGATGAAGATTGCCAACGA	TGCTGCCATCAGACAATAGC
CG7712	GTACCGCCAGATTCCCTACA	GCCCTGGATGAATTTTGAGA
CG4769	AAGGGCGGAGAGGATTACAT	GACCACTTGTGCCGCTTAAT
CG2968	CAAATCGATGTGCCTTCCTT	TCTATTCGGCAGCCTTGACT
BiP	ATGGTCCGACACCAATGTGCA	CCCAAATGAGTATCACCGTTG
Atf4	AGACGCTGCTTCGCTTCCTTC	GCCCGTAAGTGCGAGTACGCT
GFP	CGACCACTACCAGCAGAACA	GTAGAATCGAGACCGAGGAGAG

qPCR, quantitative polymerase chain reaction.

### Plasmids and transfection

For Atf4 overexpression, we cloned the *D. melanogaster* Atf4 ORF downstream of the metallothionein promoter and 5′UTR, using the parent commercial plasmid pMT/V5-HisC (Invitrogen). For promoter reporter constructs, we amplified the promoter regions of *Ldh*, *Pfk*, and *Ald* from S2 cell genomic DNA and subcloned into a vector expressing enhanced green fluorescent protein described previously (Hollien and Weissman 2006). To examine the effect of the Atf4 binding sites on the regulation these reporters, we introduced point mutations [for cyclic AMP response element (CRE)] or deleted the entire Atf4 binding motif using sequential PCR.

We transfected S2 cells with 2 μg of plasmid using Cellfectin II (Invitrogen). For polyclonal stable cell lines (as in Figure 3D and Figure 5A), we cotransfected our expression plasmids (1.8 μg) with a hygromycin or puromycin resistance plasmid (0.2 μg) and selected for resistant cells. For Atf4 overexpression studies (Figure 3D), we induced expression with CuSO_4_ (250 μM, 36 hr) before collecting RNA samples. For promoter constructs, we treated control cells transfected with pMT-GFP with CuSO_4_ (250 μM, 16 hr) before inducing ER stress with DTT as described above. Finally, for transiently transfected cells (Figure 5B), we allowed cells to recover overnight before inducing ER stress.

### Fly strains, maintenance, and ER stress induction

We raised *Drosophila melanogaster* lines on standard medium at room temperature (~22°) unless otherwise stated. Fly stocks were as follows: w*^1118^*, *hsp70*-GAL4 (*hs-GAL4*) and y[1] v[1]; P{y[+t7.7] v[+t1.8]=TRiP.JF02007}attP2 (*UAS-Atf4^RNAi^*). The *crc* (*cryptocephal*) gene encodes the *Drosophila* homolog of vertebrate Atf4 (Wu *et al.* 2007; Hewes *et al.* 2000). We obtained all fly stocks from the Bloomington Fly Stock Center (note that there was only one *Atf4* RNAi strain available). To stress flies, we grew 2- to 3-d-old males on a solid medium containing 1.3% agarose, 1% sucrose with or without Tm (10 μg/mL) for 23 hr, as described previously (Chow *et al.* 2013).

To generate *Atf4* RNAi knockdown flies, we crossed virgin *UAS- Atf4^RNAi^* females to male *hs-GAL4*. To prevent leaky expression of GAL4 from the *heat shock* promoter, we kept the progeny at 18° until feeding them with or without Tm at room temperature as 2-d-old adults.

### Lactate and oxygen consumption measurements

We plated S2 cells at 1.6 × 10^6^ cells per well in 6-well plates in media with or without Tm (5 μg/mL, 23.5 hr). Before collecting the conditioned media from the S2 cells, we counted the number of viable cells by trypan blue (0.4%) exclusion staining. The concentration of lactate produced by cells was determined using the Lactate Colorimetric Assay Kit II (BioVision) according to the manufacturer’s protocol. We measured the absorbance at 450 nm on a Synergy MX microplate reader (Biotek). Then, we divided lactate concentration by the number of cells.

For lactate measurements of flies, we homogenized at least 50 decapitated flies for each condition in PBS using a pestle. The decapitation was necessary to prevent the eye color in transgenic flies from interfering with the colorimetric lactate assay. We removed an aliquot of the homogenate and quantified total protein using the Pierce BCA protein Assay Kit (Thermo). For the remainder we heat-inactivated at 65° for 15 min and determined lactate levels as described above. We corrected the absorbance value for background by subtracting the value of same sample incubated without enzyme mix. The amount of lactate in fly samples was normalized to their protein content.

We measured the levels of dissolved oxygen in cell cultures for 5 min using Clark-type oxygen electrodes (YSI Inc.). We plotted oxygen concentration *vs.* time, and fit the data to a line to obtain the oxygen consumption rate.

## Results

### ER stress induces a coordinated change in metabolic gene expression in S2 cells

**Figure 1:**
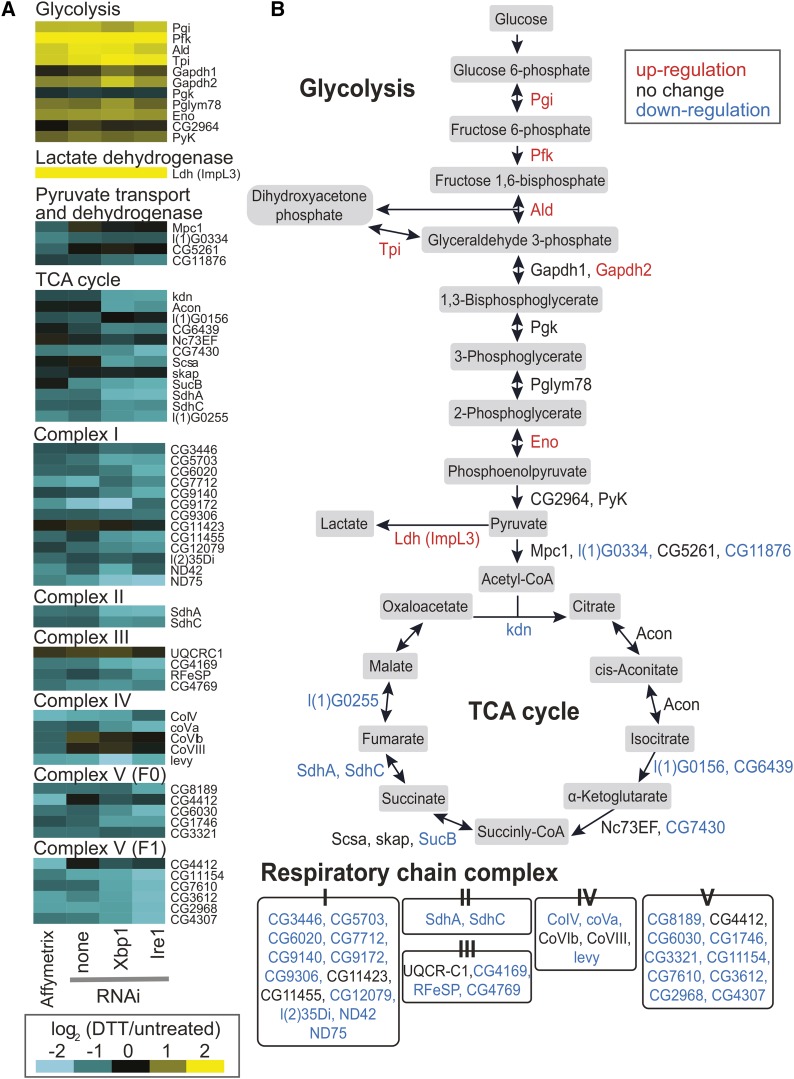
Microarray analysis reveals a coordinated change in the expression of metabolic genes in S2 cells treated with dithiothreitol (DTT). (A) We analyzed the expression of genes in glycolysis, the tricarboxylic acid (TCA) cycle, and the respiratory chain complex using previously published microarray data (Hollien and Weissman 2006). Briefly, S2 cells were incubated with and without DTT (2 mM, 7 hr), and relative RNA levels were measured by Affymetrix microarrays. In parallel, S2 cells were depleted of the indicated factors by RNA interference, then incubated with and without DTT (2 mM, 7.4 hr). Relative RNA levels were then measured by spotted microarray. Further details and complete data are available at the Gene Expression Omnibus, accession GPL3781 (http://www.ncbi.nlm.nih.gov/geo/). (B) Schematic diagram of glucose metabolism with expression data from (A). Red indicates mRNA levels were up-regulated at least 2-fold by DTT, blue indicates down-regulation by at least 1.5-fold, and black indicates changes between these thresholds.

To explore the regulation of metabolism during ER stress, we examined gene expression patterns from our previously published microarray studies of *D. melanogaster* S2 cells (Hollien and Weissman 2006). We found that the mRNA levels of most enzymes involved in central carbon metabolism changed when cells were treated with DTT (2 mM), a reducing agent that induces ER stress by disrupting disulfide bond formation within the ER. In response to DTT, genes encoding glycolytic enzymes were up-regulated whereas genes encoding TCA cycle enzymes and the respiratory chain complexes were down-regulated (Figure 1 and Supporting Information, Table S1). In addition, expression of *Lactate dehydrogenase* (*Ldh*, also known as *ImpL3* and *CG10160*), which codes for the enzyme that converts pyruvate to lactate, was dramatically increased. These changes in metabolic gene expression suggest a shift in glucose metabolism from OXPHOS to glycolysis.

**Figure 2:**
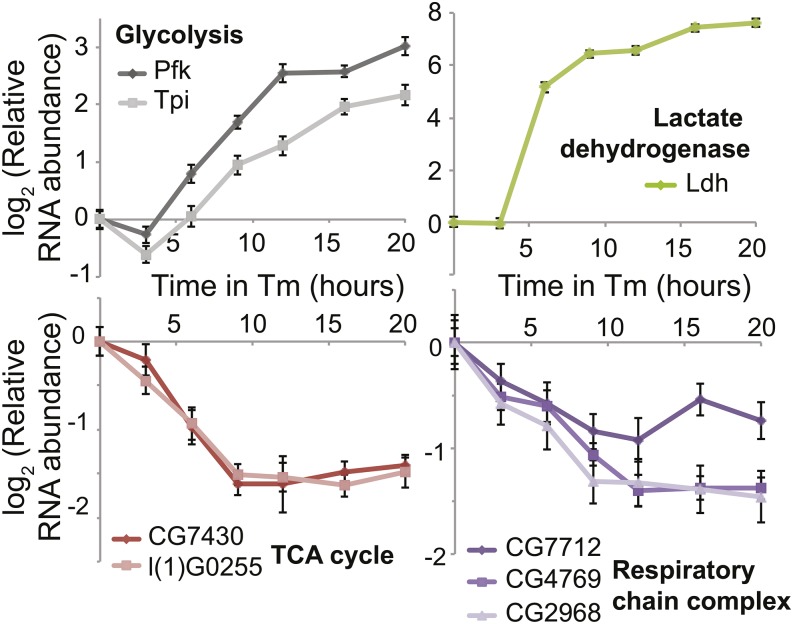
Metabolic gene expression is regulated by Tm in S2 cells. We treated S2 cells with Tm (5 μg/mL) and measured relative RNA levels by quantitative polymerase chain reaction. We normalized all RNA measurements to the RNA levels of *Ribosomal protein L19 (RpL19)*. Data are presented as means ± SEs of three technical replicates, and are representative of two independent experiments. *Pfk*, *phosphofructokinase*; TCA, tricarboxylic acid; Tm, tunicamycin; *Tpi*, *triosephosphate isomerase*.

To confirm these findings and to determine whether the expression changes were specific to DTT or a general response to ER stress, we treated S2 cells with another ER stress reagent, Tm (5 μg/mL), which inhibits *N*-linked glycosylation. We then monitored RNA levels of several genes over time, by qPCR (Figure 2). Consistent with the microarray results, expression of glycolytic genes and *Ldh* was increased and expression of genes encoding TCA cycle enzymes and respiratory chain complexes was decreased in response to Tm.

### Atf4 regulates glycolytic genes and *Ldh* during ER stress

**Figure 3:**
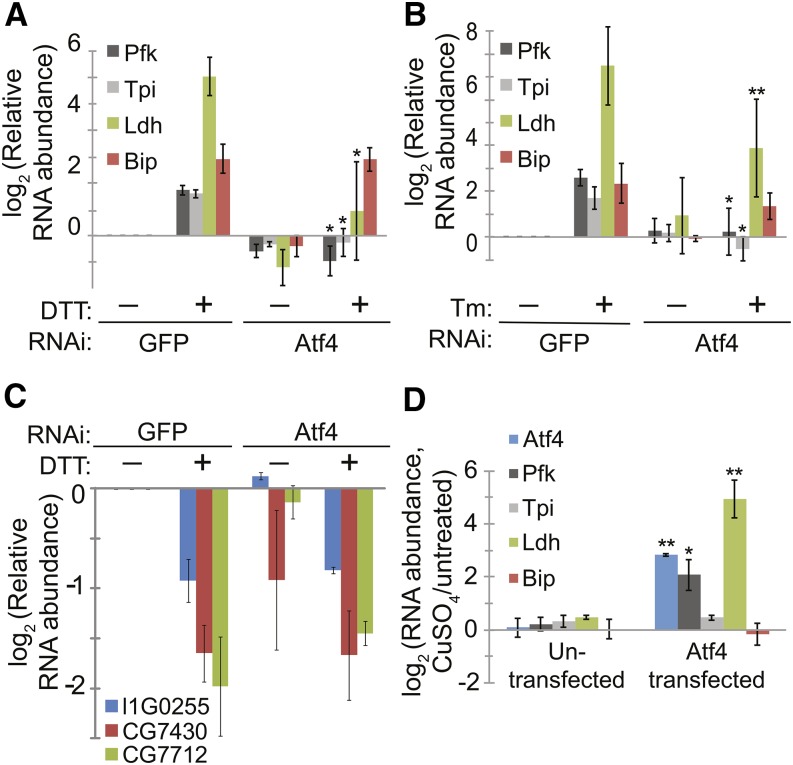
Atf4 is necessary and sufficient for up-regulation of glycolytic genes and Ldh. (A−B) We incubated S2 cells with dsRNA targeting either GFP (as a negative control) or Atf4, allowed cells to recover, and incubated with and without either DTT (2 mM, 6 hr, A) or Tm (5 μg/mL, 16 hr, B). We then measured the relative RNA levels for the indicated genes by qPCR. *P < 0.05; **P < 0.005 *vs.* DTT or Tm-treated control cells (GFP RNAi), Student’s paired *t*-test. (C) Samples from (A) were analyzed for relative mRNA levels of TCA cycle and respiratory chain complex genes, by qPCR. (D) We stably transfected S2 cells with a plasmid expressing Atf4 under the control of a copper-inducible promoter. We incubated both untransfected and stable cell lines with and without copper (250 μM, 36 hr), then measured mRNA levels by qPCR. *P < 0.05; **P < 0.005 *vs.* untransfected control cells, student’s paired *t*-test. For all panels, data are presented as means ± SDs of 3 independent experiments. Atf4, activating transcription factor 4; dsRNA, double-stranded RNA; DTT, dithiothreitol; GFP, green fluorescent protein; qPCR, quantitative polymerase chain reaction; RNAi, RNA interference; TCA, tricarboxylic acid; Tm, tunicamycin

We next investigated which branch of the UPR signaling network is responsible for the regulation of metabolic genes during ER stress. Our array data showed that this regulation was not dependent on Ire1 or Xbp1, as depleting either of these factors by RNAi had little effect on the regulation of the metabolic genes examined (Figure 1A). We therefore tested the role of Atf4 (also known as crc), the other main transcription factor known to mediate the UPR in flies (Ryoo and Steller 2007). Targeting Atf4 in S2 cells by RNAi resulted in a reduction in Atf4 mRNA levels to 16% ± 10% compared with control cells. Knockdown of Atf4 did not affect up-regulation of *BiP* (also known as *Hsp70-3* and *CG4147*), a major ER chaperone whose up-regulation during ER stress is dependent on the Ire1-Xbp1 branch of UPR in S2 cells (Moore *et al.* 2013) (Figure 3, A and B), nor did it significantly affect the down-regulation of TCA cycle and respiratory chain complex genes (Figure 3C). However, Atf4 knockdown completely blocked induction of *Phosphofructokinase (Pfk)*, *Triosephosphate isomerase (Tpi)*, and *Ldh* by both DTT (2 mM, 6 hr) and Tm (5 μg/mL, 16 hr) (Figure 3, A and B), indicating a specific role for Atf4 in regulating glycolytic genes.

Next, we asked whether Atf4 is sufficient to up-regulate the expression of glycolytic genes. To overexpress Atf4, we stably transfected S2 cells with a plasmid expressing *D. melanogaster* Atf4 under the control of the copper-inducible metallothionein promoter. This plasmid did not include the natural 5′UTR of Atf4, whose upstream ORFs would prevent substantial translation in the absence of ER stress, but rather used the metallothionein 5′UTR. Upon addition of copper sulfate to the media (250 μM CuSO_4_, 36 hr), transfected cells (but not untransfected control cells) displayed an increase in the expression of *Pfk* and *Ldh* (Figure 3D), similar to that induced by ER stress (Figure 3, A and B). *Tpi* mRNA levels were unchanged, however, suggesting that Atf4 is sufficient for the up-regulation of a subset of the ER stress-regulated glycolytic genes. We confirmed that overexpression of Atf4 did not cause ER stress *per se* by measuring BiP mRNA levels, which were unchanged in response to CuSO_4_ (Figure 3D). Taken together, these results indicate that Atf4 is necessary for the up-regulation of glycolytic genes and *Ldh* during ER stress, and is sufficient for the up-regulation of at least two of these genes.

### Atf4 binding sites in the *Ldh* and *Pfk* promoters are important for regulation by ER stress

**Figure 4:**
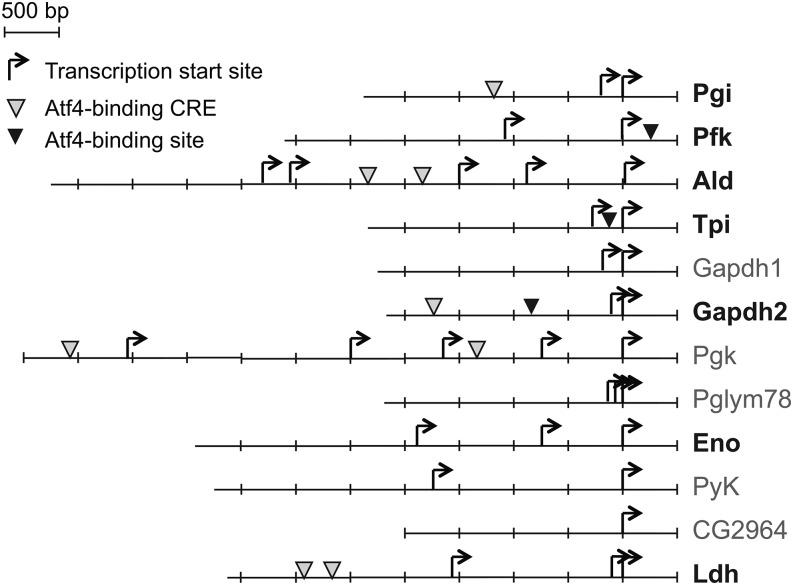
Potential Atf4 binding sites are found in the promoters of glycolytic genes and *Ldh*. Schematic representations of the location of putative Atf4 binding sites are shown. Arrows indicate transcription start sites as annotated in FlyBase, and arrowheads indicate consensus Atf4 binding CREs (TGACGT; gray) and the Atf4 binding motif identified in mouse embryonic fibroblasts (Han *et al.* 2013) (TT(G/T)CATCA(G/T); black). We examined 2 kb upstream and 0.5 kb downstream of all transcription start sites. Genes whose expression was up-regulated by twofold or greater by DTT are indicated in bold (see Figure 1); others were not changed during ER stress. Atf4, activating transcription factor 4; DTT, dithiothreitol; ER, endoplasmic reticulum; *Ldh*, *Lactate dehydrogenase*.

Atf4 is a member of the bZIP family of transcription factors, which regulate target genes through the CRE (TGACGT) (Lin and Green 1988). A recent chromatin immunoprecipitation-RNA sequencing study also identified the TT(G/T)CATCA(G/T) motif as an Atf4 binding site in mouse embryonic fibroblasts (Han *et al.* 2013). We examined the promoter regions of glycolytic genes in *D. melanogaster* (2 kb upstream and 0.5 kb downstream of the annotated transcription start sites) and found that six of the seven glycolytic genes up-regulated by ER stress contained at least one of these Atf4 binding sites. Conversely, only one of the four glycolytic genes not significantly up-regulated by ER stress contained an Atf4 binding site (Figure 4).

**Figure 5:**
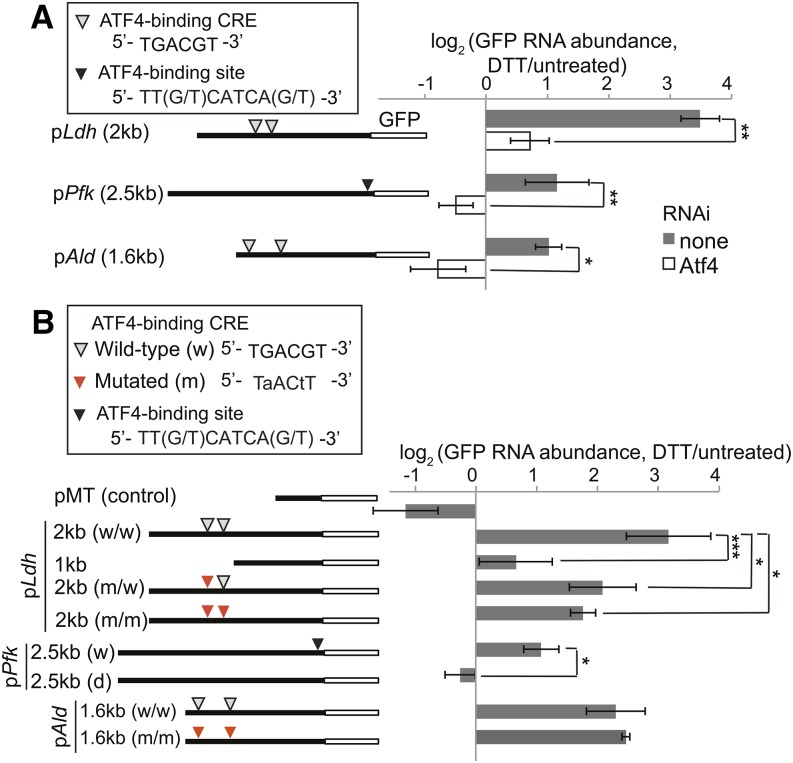
Atf4 binding sites within the promoters of Ldh and Pfk mediate Atf4-dependent transcriptional up-regulation. (A) We stably transfected S2 cells with the GFP reporter constructs diagrammed on the left. We then mock-treated or depleted cells of Atf4 by RNAi, incubated with and without DTT (2 mM, 5 hr), and measured relative GFP RNA levels by qPCR. (B) We transiently transfected S2 cells with reporter constructs containing wild-type or mutated promoter sequences as shown on the left. We then incubated cells with or without DTT (2 mM, 5 hr) and measured relative GFP RNA levels by qPCR. For all panels: shown are the means ± SDs of at least three independent experiments. *P < 0.05; **P < 0.005; ***P < 0.001, Student’s paired *t*-test. Atf4, activating transcription factor 4; dsRNA, double-stranded RNA; DTT, dithiothreitol; GFP, green fluorescent protein; *Ldh*, *Lactate dehydrogenase*; *Pfk*, *phosphofructokinase*; qPCR, quantitative polymerase chain reaction; RNAi, RNA interference; TCA, tricarboxylic acid; Tm, tunicamycin

To investigate the importance of these Atf4 binding sites in up-regulating glycolytic genes during ER stress, we took a reporter-based approach. *Ldh*, the most highly up-regulated gene involved in the metabolic pathways studied here, contains two CREs within 2 kb upstream of its transcription start site (Figure 4). We made reporter constructs containing different lengths of the promoter region of *Ldh*, followed by the coding sequence for green fluorescent protein (GFP) (Figure 5). We transfected S2 cells with these p*Ldh*-GFP plasmids, incubated cells with and without DTT (2 mM, 5 hr), and measured the expression levels of GFP mRNA by qPCR. As a negative control, we used a plasmid expressing GFP under the control of the metallothionein promoter, and added CuSO_4_ to cells (250 μM, 16 hr) before the addition of DTT.

We found that when we included 2 kb upstream of the transcription start site for *Ldh* in our reporter, GFP mRNA was up-regulated 12-fold during ER stress, in an Atf4 dependent manner (Figure 5A). However, when we included only 1 kb of the promoter, a region that lacks the Atf4 binding sites described above, GFP mRNA levels were not significantly changed during ER stress (Figure 5B). To test the importance of the CRE sites more specifically, we introduced into each CRE two point mutations that had been previously shown to abolish Atf4 binding in mammalian cells (Bouman *et al.* 2010). Mutating either the upstream CRE or both of the CREs in the 2 kb promoter partially blocked its regulation during ER stress (Figure 5B).

Some glycolytic genes in flies, including *Pfk*, lack CREs but contain the TT(G/T)CATCA(G/T) motif (Figure 4). To test the importance of this motif, we constructed a reporter containing 2.5 kb of the *Pfk* promoter, followed by the coding sequence for GFP. Cells stably transfected with p*Pfk*-GFP showed increased levels of GFP mRNA during ER stress, an effect that was abolished when Atf4 was depleted by RNAi (Figure 5A). Furthermore, deleting the TTGCATCAG motif in this reporter blocked GFP up-regulation by ER stress (Figure 5B).

Together, our results support a model where Atf4 regulates the expression of glycolytic genes by binding to known motifs within their promoters. However, this was not true for every promoter we tested: repeating the above reporter-based assay using 1.6 kb upstream of the *Aldolase* (*Ald*) transcription start site resulted in clear Atf4-dependent up-regulation of GFP during ER stress (Figure 5A), but mutation of the 2 CREs within this reporter had no effect on its regulation (Figure 5B). These results open up the possibility that Atf4 also regulates glycolytic genes through alternative binding sites (Fawcett *et al.* 1999; Gombart *et al.* 2007; Gjymishka *et al.* 2008; Kode *et al.* 2012; Chiang *et al.* 2013; Han *et al.* 2013) or via both direct and indirect mechanisms.

### *Ldh* expression and lactate levels are increased in *Drosophila melanogaster* exposed to ER stress

**Figure 6:**
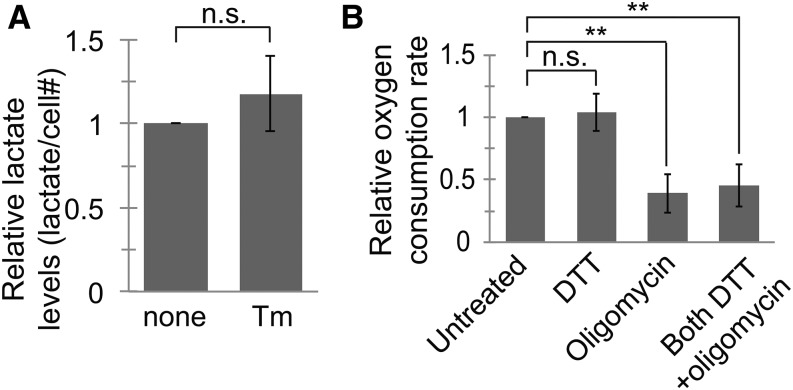
Lactate production and oxygen consumption are not changed in S2 cells during ER stress. (A) We measured lactate levels in the culture media of S2 cells treated with or without Tm (5 μg/mL, 23 hr). Although DTT is a typically more potent inducer of ER stress in these cells, its function as a reducing agent was incompatible with the redox-based lactate assay. We normalized the lactate concentration by number of cells and to the untreated samples. (B) We incubated S2 cells with or without DTT (2 mM, 5 hr), then added +/− oligomycin (1 μg/mL) for 10 min. We then measured the oxygen consumption rate of cells and normalized to the rate for untreated cells. For both panels, data are presented as means ± SDs of at least 3 independent experiments. ** *P* < 0.005, Student’s paired *t*-test. DTT, dithiothreitol; ER, endoplasmic reticulum; n.s., not significant; Tm, tunicamycin.

The metabolic gene expression changes described above suggest a switch from an OXPHOS-based metabolic state to a more glycolytic one. Because OXPHOS is the main oxygen-consuming process in most cells, such a shift would be characterized by reduced oxygen consumption and an increase in lactate production, as pyruvate is converted to lactate rather than imported into the mitochondria (Wu *et al.* 2007). Surprisingly, neither oxygen consumption nor lactate concentration in the culture media was changed upon treatment of S2 cells with ER stress-inducing agents (Figure 6). Because this lack of effect might be due to the *in vitro* nature of the experimental system, we decided to test whether metabolic gene expression changes occur *in vivo* and whether they are mirrored by changes in actual metabolism.

**Figure 7:**
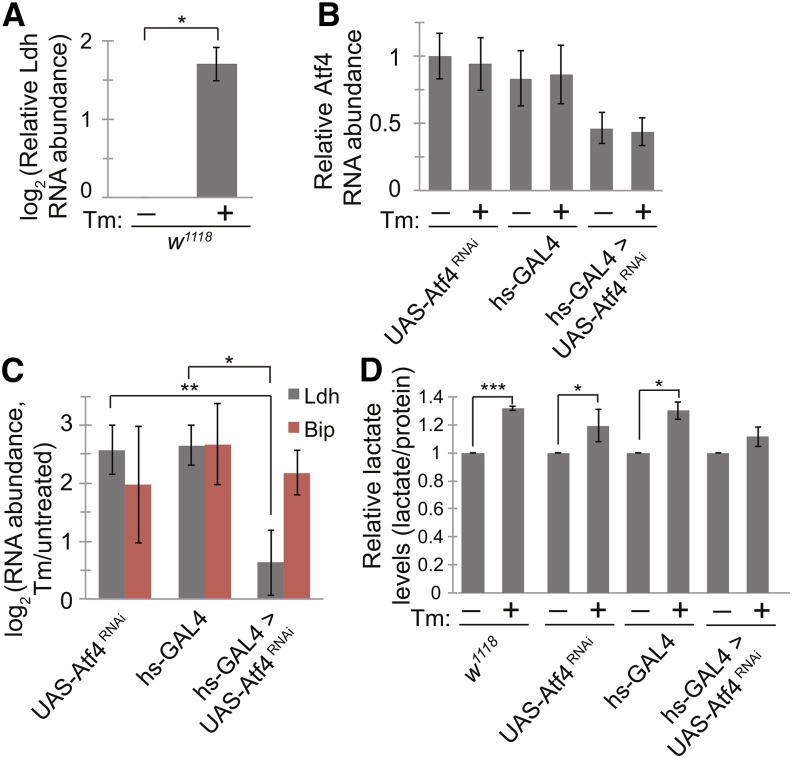
Flies display metabolic changes during ER stress *in vivo*. (A) We fed male *D. melanogaster* w1118 with Tm (10 μg/mL, 23 hr) to induce ER stress and measured *Ldh* mRNA levels by qPCR. (B−C) We crossed *UAS-Atf4^RNAi^* to hs-GAL4 to obtain Atf4 knockdown flies. We stressed each strain of flies as in (A) and compared the RNA levels of *Atf4* (B), *Ldh*, and *BiP* (C) by qPCR. (D) We measured lactate levels in extracts from *D. melanogaster* fed with or without Tm as in A. Lactate concentrations were normalized using total protein concentrations. For all panels: data are presented as means ± SDs of 3 independent experiments. *P < 0.05; **P < 0.005; ***P < 0.001, Student’s paired *t*-test. Atf4, activating transcription factor 4; ER, endoplasmic reticulum; *Ldh*, *Lactate dehydrogenase*; qPCR, quantitative polymerase chain reaction; Tm, tunicamycin.

To induce ER stress in flies, we fed *Drosophila melanogaster* strain *w^1118^* media containing Tm (10 μg/mL) for 23 hr. We then isolated RNA from flies and measured the mRNA levels for several genes by qPCR. Consistent with our findings in S2 cells, flies fed Tm showed increased *Ldh* expression (Figure 7A). Levels of other glycolytic genes, however, were not significantly changed in the presence of Tm. Because *Ldh* was the most strongly up-regulated metabolic gene in S2 cells (~100 fold), and was regulated to a much lesser extent in flies (~3 fold), it is possible that this regulation occurs only in certain tissues, resulting in expression changes for other genes that were below our detection limit when whole flies were assayed.

The *Atf4* gene in flies is essential for normal development; null mutants for *Atf4* are lethal (Hewes *et al.* 2000). To examine whether *Ldh* regulation by ER stress in flies is dependent on Atf4, we therefore used the GAL4/UAS system to inducibly knockdown *Atf4* expression by RNAi. We crossed a *heatshock GAL4 (hs-GAL4)* line to a *UAS-Atf4^RNAi^*. We then heat-shocked (37°, 45 min) the progeny of these crosses (*hs-GAL4 > UAS-Atf4^RNAi^*) at various times during development, but did not detect any changes in adult *Atf4* expression by qPCR. However, we saw an approximately 50% reduction of Atf4 in *hs-GAL4 > UAS-Atf4^RNAi^* flies, compared to either parental strain, when they were continuously grown at 18° and then shifted to RT (22°) and treated with or without Tm (10 μg/mL, 23 hr) (Figure 7B). Flies depleted of Atf4 displayed a significant decrease in the up-regulation of *Ldh* during ER stress relative to the parental strains (Figure 7C). The up-regulation of BiP, in contrast, was consistent across all fly lines (Figure 7C). Thus, Atf4 up-regulates *Ldh* in flies during ER stress.

To test whether the increased expression of *Ldh* resulted in increased lactate production in response to ER stress, we measured lactate levels in extracts from flies incubated with and without Tm. Flies exposed to ER stress exhibited a ~30% increase in lactate compared with control flies, suggesting a metabolic shift to glycolysis (Figure 7D). Although analysis of lactate levels in flies depleted of Atf4 suggested that this effect was dependent on Atf4, the changes especially in *UAS-Atf4^RNAi^* flies were too small to allow for detection of significant differences between parental and progeny strains (Figure 7D). Taken together, these results suggest that flies subjected to ER stress up-regulate *Ldh* expression in an Atf4-dependent manner, and that unlike in S2 cells, this regulation results in a metabolic shift evidenced by the accumulation of lactate.

## Discussion

We have shown that *D. melanogaster* S2 cells subjected to ER stress up-regulate glycolytic genes and *Ldh* and down-regulate genes involved in the TCA cycle and respiratory chain complex (Figure 1 and Figure 2). Furthermore, Atf4 is responsible for the up-regulation of glycolytic genes and *Ldh* (Figure 3 and Figure 7). How TCA cycle and respiratory chain complex genes are down-regulated during ER stress requires further investigation, although the lack of effect of Atf4 depletion (Figure 3C) suggests that these are not regulated as an indirect consequence of glycolysis up-regulation.

Despite a highly coordinated change in gene expression for metabolic genes during ER stress, we did not detect any changes in actual metabolism in S2 cells (Figure 6). Because these cells have been in culture for decades and have likely been selected for rapid proliferation, it is possible that they are already undergoing some version of aerobic glycolysis, such that the underlying gene regulation during ER stress is preserved but any metabolic changes are masked. Others have also noted that S2 cells are resistant to hypoxia, and do not produce more lactate except in extreme conditions (Swiech *et al.* 2008). The increase in lactate observed through *in vivo* studies in flies subjected to ER stress (Figure 7D), however, suggests that in a more physiological setting, the gene expression changes shown here do mediate a metabolic shift toward aerobic glycolysis.

Up-regulation of glycolytic genes during ER stress has not been observed in genome-wide studies of mammalian cells (Marciniak *et al.* 2004; Hollien *et al.* 2009; Han *et al.* 2013). However, several lines of evidence suggest that mammalian cells subjected to ER stress may undergo a glycolytic shift. For example, a recent study examining human gliomas found coordinated up-regulation of UPR targets and glycolysis, which correlated with poor patient prognosis (Epple *et al.* 2013); and both ER stress (Win *et al.* 2013) and overexpression of Perk (Muñoz *et al.* 2013) have been shown to reduce mitochondrial respiration in cultured mammalian cells.

The link between ER stress and metabolism can be rationalized by the need to generate building blocks for biosynthesis of glycoproteins and lipids. Early intermediates of glycolysis are necessary for production of uridine diphosphate-*N*-acetylglucosamine (UDP-GlcNAc), an important donor molecule for *N*-glycosylation of proteins in the ER. Both fructose-6-phosphate and dihydroxyacetone phosphate also are required for synthesis of glycolipids. An increased flux through glycolysis may therefore be important to support the increased production of glycerophospholipids and glycoproteins that are associated with the UPR (Sriburi 2004; Sriburi *et al.* 2006; Lee *et al.* 2008). In support of this view, glucose deprivation or inhibition of glycolysis with 2-deoxy-D-glucose induces the UPR, which contributes to cell death, especially in cancer cells (Liu *et al.* 2009; Xi *et al.* 2011; Palorini *et al.* 2013), and this effect can be rescued by UDP-GlcNAc (Palorini *et al.* 2013). The hexosamine biosynthetic pathway generating UDP-GlcNAc is also directly activated by Xbp1 (Denzel *et al.* 2014; Wang *et al.* 2014), stimulates cardioprotection during ischemia/reperfusion injury (Wang *et al.* 2014), and increases longevity in worms (Denzel *et al.* 2014).

A second, nonmutually exclusive explanation for a shift to glycolysis during ER stress is the need to limit production of ROS. Along with mitochondrial respiration, protein folding in the ER is one of the main sources of ROS (Solaini *et al.* 2010), which are produced by the normal process of disulfide bond-coupled folding (Higa and Chevet 2012). If allowed to accumulate, these ROS can cause oxidative stress and damage to cells, eventually leading to apoptosis. Several studies have confirmed that ROS are produced during ER stress (Harding *et al.* 2003; Cullinan and Diehl 2006; Tavender and Bulleid 2010), when protein folding is inefficient and more rounds of oxidation and reduction are required to fold proteins. Limiting other sources of oxidative stress, such as by down-regulating the TCA cycle and thereby restricting the flux through OXPHOS (the main source of ROS in the mitochondria), may be a way to mitigate the damage and allow cells to recover more effectively.

Finally, the advantage of the Warburg effect for tumor growth may arise from the increased rate of ATP production by glycolysis compared to OXPHOS, despite its lower efficiency of conversion (Pfeiffer *et al.* 2001). By analogy, a metabolic shift during ER stress could rapidly supply ATP necessary for protein folding and processing. Indeed, cancer cells showing elevated levels of ENTPD5, an ER UDPase, promotes aerobic glycolysis to increase ATP for protein *N*-glycosylation and refolding (Fang *et al.* 2010; Shen *et al.* 2011).

Overall, our results identify Atf4 as a transcriptional regulator of glycolysis during ER stress. As *Atf4* is expressed throughout fly development (Hewes *et al.* 2000), it may regulate glycolysis in other situations as well: notably, *Atf4* mutant flies are lean and have reduced circulating carbohydrates, suggesting a role in metabolism (Seo *et al.* 2009). Furthermore, because the Perk-Atf4 branch of UPR is activated during hypoxia (Blais *et al.* 2004), it will be interesting to see whether Atf4 contributes to regulation of glycolysis in other developmental, physiological (hypoxia), or pathological process during which glycolysis regulated. More broadly, because the UPR is activated in many types of cancer, its ability to regulate glucose metabolism may play a contributing role in the Warburg effect.

## 

## Supplementary Material

Supporting Information

## References

[bib1] Basseri S. Austin R. C., 2012 Endoplasmic reticulum stress and lipid metabolism: mechanisms and therapeutic potential. Biochem. Res. Int. 2012: 1–13. 10.1155/2012/841362 PMC3238353 22195283

[bib2] Blais J. D. Filipenko V. Bi M. Harding H. P. Ron D., 2004 Activating transcription factor 4 is translationally regulated by hypoxic stress. Mol. Cell. Biol. 24: 7469–7482. 15314157 10.1128/MCB.24.17.7469-7482.2004 PMC506979

[bib3] Bouman L. Schlierf A. Lutz A. K. Shan J. Deinlein A., 2010 Parkin is transcriptionally regulated by ATF4: evidence for an interconnection between mitochondrial stress and ER stress. Cell Death Differ. 18: 769–782. 21113145 10.1038/cdd.2010.142 PMC3131924

[bib4] Calfon M. Zeng H. Urano F. Till J. H. Hubbard S. R., 2002 IRE1 couples endoplasmic reticulum load to secretory capacity by processing the XBP-1 mRNA. Nature 415: 92–96. 11780124 10.1038/415092a

[bib5] Chiang C.-K. Nangaku M. Tanaka T. Iwawaki T. Inagi R., 2013 Endoplasmic reticulum stress signal impairs erythropoietin production: a role for ATF4. Am. J. Physiol. Cell Physiol. 304: C342–C353. 23242184 10.1152/ajpcell.00153.2012

[bib6] Chow C. Y. Wolfner M. F. Clark A. G., 2013 Using natural variation in Drosophila to discover previously unknown endoplasmic reticulum stress genes. Proc. Natl. Acad. Sci. USA 110: 9013–9018. 23667151 10.1073/pnas.1307125110 PMC3670321

[bib7] Cox J. S. Shamu C. E. Walter P., 1993 Transcriptional induction of genes encoding endoplasmic reticulum resident proteins requires a transmembrane protein kinase. Cell 73: 1197–1206. 8513503 10.1016/0092-8674(93)90648-a

[bib8] Cullinan S. B. Diehl J. A., 2006 Coordination of ER and oxidative stress signaling: the PERK/Nrf2 signaling pathway. Int. J. Biochem. Cell Biol. 38: 317–332. 16290097 10.1016/j.biocel.2005.09.018

[bib9] Dang C. V., 2012 Links between metabolism and cancer. Genes Dev. 26: 877–890. 22549953 10.1101/gad.189365.112 PMC3347786

[bib10] Denzel M. S. Storm N. J. Gutschmidt A. Baddi R. Hinze Y., 2014 Hexosamine pathway metabolites enhance protein quality control and prolong life. Cell 156: 1167–1178. 24630720 10.1016/j.cell.2014.01.061

[bib11] Epple L. M. Dodd R. D. Merz A. L. Dechkovskaia A. M. Herring M., 2013 Induction of the unfolded protein response drives enhanced metabolism and chemoresistance in glioma cells. PLoS One 8: e73267. 24039668 10.1371/journal.pone.0073267 PMC3748289

[bib12] Fang M. Shen Z. Huang S. Zhao L. Chen S., 2010 The ER UDPase ENTPD5 promotes protein N-glycosylation, the Warburg effect, and proliferation in the PTEN pathway. Cell 143: 711–724. 21074248 10.1016/j.cell.2010.10.010

[bib13] Fawcett T. W. Martindale J. L. Guyton K. Z. Hai T. Holbrook N. J., 1999 Complexes containing activating transcription factor (ATF)/cAMP-responsive-element-binding protein (CREB) interact with the CCAAT/enhancer-binding protein (C/EBP)-ATF composite site to regulate Gadd153 expression during the stress response. Biochem. J. 339: 135–141. 10085237 PMC1220137

[bib14] Fox C. J. Hammerman P. S. Thompson C. B., 2005 Fuel feeds function: energy metabolism and the T-cell response. Nat. Rev. Immunol. 5: 844–852. 16239903 10.1038/nri1710

[bib15] Gaddam D. Stevens N. Hollien J., 2013 Comparison of mRNA localization and regulation during endoplasmic reticulum stress in *Drosophila cells*. Mol. Biol. Cell 24: 14–20. 23135994 10.1091/mbc.E12-06-0491 PMC3530775

[bib16] Garg A. D. Kaczmarek A. Krysko O. Vandenabeele P. Krysko D. V., 2012 ER stress-induced inflammation: does it aid or impede disease progression? Trends Mol. Med. 18: 589–598. 22883813 10.1016/j.molmed.2012.06.010

[bib17] Gjymishka A. Palii S. S. Shan J. Kilberg M. S., 2008 Despite increased ATF4 binding at the C/EBP-ATF composite site following activation of the unfolded protein response, system A transporter 2 (SNAT2) transcription activity is repressed in HepG2 cells. J. Biol. Chem. 283: 27736–27747. 18697751 10.1074/jbc.M803781200 PMC2562058

[bib18] Gombart A. F. Grewal J. Koeffler H. P., 2007 ATF4 differentially regulates transcriptional activation of myeloid-specific genes by C/EBPepsilon and C/EBPalpha. J. Leukoc. Biol. 81: 1535–1547. 17347301 10.1189/jlb.0806516

[bib19] Han J. Back S. H. Hur J. Lin Y.-H. Gildersleeve R., 2013 ER-stress−induced transcriptional regulation increases protein synthesis leading to cell death. Nat. Cell Biol. 15: 481–490. 23624402 10.1038/ncb2738 PMC3692270

[bib20] Harding H. P. Zhang Y. Ron D., 1999 Protein translation and folding are coupled by an endoplasmic-reticulum-resident kinase. Nature 397: 271–274. 9930704 10.1038/16729

[bib21] Harding H. P. Novoa I. Zhang Y. Zeng H. Wek R., 2000 Regulated translation initiation controls stress-induced gene expression in mammalian cells. Mol. Cell 6: 1099–1108. 11106749 10.1016/s1097-2765(00)00108-8

[bib22] Harding H. P. Zhang Y. Zeng H. Novoa I. Lu P. D., 2003 An integrated stress response regulates amino acid metabolism and resistance to oxidative stress. MOLCEL 11: 619–633. 10.1016/s1097-2765(03)00105-9 12667446

[bib23] Haze K. Yoshida H. Yanagi H. Yura T. Mori K., 1999 Mammalian transcription factor ATF6 is synthesized as a transmembrane protein and activated by proteolysis in response to endoplasmic reticulum stress. Mol. Biol. Cell 10: 3787–3799. 10564271 10.1091/mbc.10.11.3787 PMC25679

[bib24] Hewes R. S. Schaefer A. M. Taghert P. H., 2000 The cryptocephal gene (ATF4) encodes multiple basic-leucine zipper proteins controlling molting and metamorphosis in *Drosophila*. Genetics 155: 1711–1723. 10924469 10.1093/genetics/155.4.1711 PMC1461179

[bib25] Higa A. Chevet E., 2012 Redox signaling loops in the unfolded protein response. Cell. Signal. 24: 1548–1555. 22481091 10.1016/j.cellsig.2012.03.011

[bib26] Hollien J., 2013 Evolution of the unfolded protein response. Biochim. Biophys. Acta. 1833: 2458–2463. 23369734 10.1016/j.bbamcr.2013.01.016

[bib27] Hollien J. Weissman J. S., 2006 Decay of endoplasmic reticulum-localized mRNAs during the unfolded protein response. Science 313: 104–107. 16825573 10.1126/science.1129631

[bib28] Hollien J. Lin J. H. Li H. Stevens N. Walter P., 2009 Regulated Ire1-dependent decay of messenger RNAs in mammalian cells. J. Cell Biol. 186: 323–331. 19651891 10.1083/jcb.200903014 PMC2728407

[bib29] Kode A. Mosialou I. Silva B. C. Joshi S. Ferron M., 2012 FoxO1 protein cooperates with ATF4 protein in osteoblasts to control glucose homeostasis. J. Biol. Chem. 287: 8757–8768. 22298775 10.1074/jbc.M111.282897 PMC3308768

[bib30] Lee A.-H. Scapa E. F. Cohen D. E. Glimcher L. H., 2008 Regulation of hepatic lipogenesis by the transcription factor XBP1. Science 320: 1492–1496. 18556558 10.1126/science.1158042 PMC3620093

[bib31] Li Y. Padmanabha D. Gentile L. B. Dumur C. I. Beckstead R. B., 2013 HIF- and non-HIF-regulated hypoxic responses require the estrogen-related receptor in *Drosophila melanogaster*. PLoS Genet. 9: e1003230. 23382692 10.1371/journal.pgen.1003230 PMC3561118

[bib32] Lin Y. S. Green M. R., 1988 Interaction of a common cellular transcription factor, ATF, with regulatory elements in both E1a- and cyclic AMP-inducible promoters. Proc. Natl. Acad. Sci. USA 85: 3396–3400. 2835770 10.1073/pnas.85.10.3396 PMC280216

[bib33] Liu H. Jiang C. C. Lavis C. J. Croft A. Dong L., 2009 2-Deoxy-D-glucose enhances TRAIL-induced apoptosis in human melanoma cells through XBP-1-mediated up-regulation of TRAIL-R2. Mol. Cancer 8: 122. 20003459 10.1186/1476-4598-8-122 PMC2803449

[bib34] Logue S. Cleary P. Saveljeva S. Samali A., 2013 New directions in ER stress-induced cell death. Apoptosis 18: 537–546. 23430059 10.1007/s10495-013-0818-6

[bib35] Marciniak S. J. Yun C. Y. Oyadomari S. Novoa I. Zhang Y., 2004 CHOP induces death by promoting protein synthesis and oxidation in the stressed endoplasmic reticulum. Genes Dev. 18: 3066–3077. 15601821 10.1101/gad.1250704 PMC535917

[bib36] Moore K. A. Hollien J., 2012 The unfolded protein response in secretory cell function. Annu. Rev. Genet. 46: 165–183. 22934644 10.1146/annurev-genet-110711-155644

[bib37] Moore K. A. Plant J. J. Gaddam D. Craft J. Hollien J., 2013 Regulation of sumo mRNA during endoplasmic reticulum stress. PLoS ONE 8: e75723. 24058701 10.1371/journal.pone.0075723 PMC3776770

[bib38] Mori K. Ma W. Gething M. J. Sambrook J., 1993 A transmembrane protein with a cdc2+/CDC28-related kinase activity is required for signaling from the ER to the nucleus. Cell 74: 743–756. 8358794 10.1016/0092-8674(93)90521-q

[bib39] Muñoz J. P. Ivanova S. Sánchez-Wandelmer J. Martínez-Cristóbal P. Noguera E., 2013 Mfn2 modulates the UPR and mitochondrial function via repression of PERK. EMBO J. 32: 2348–2361. 23921556 10.1038/emboj.2013.168 PMC3770335

[bib40] Palorini R. Cammarata F. P. Cammarata F. Balestrieri C. Monestiroli A., 2013 Glucose starvation induces cell death in K-ras-transformed cells by interfering with the hexosamine biosynthesis pathway and activating the unfolded protein response. Cell Death Dis. 4: e732. 23868065 10.1038/cddis.2013.257 PMC3730427

[bib41] Pfeiffer T. Schuster S. Bonhoeffer S., 2001 Cooperation and competition in the evolution of ATP-producing pathways. Science 292: 504–507. 11283355 10.1126/science.1058079

[bib42] Rafalski V. A. Mancini E. Brunet A., 2012 Energy metabolism and energy-sensing pathways in mammalian embryonic and adult stem cell fate. J. Cell Sci. 125: 5597–5608. 23420198 10.1242/jcs.114827 PMC3575699

[bib43] Ryoo H. D. Steller H., 2007 Unfolded protein response in *Drosophila*: why another model can make it fly. Cell Cycle 6: 830–835. 17387279 10.4161/cc.6.7.4064

[bib44] Seo J. Fortuno E. S. Suh J. M. Stenesen D. Tang W., 2009 Atf4 regulates obesity, glucose homeostasis, and energy expenditure. Diabetes 58: 2565–2573. 19690063 10.2337/db09-0335 PMC2768187

[bib45] Shen Z. Huang S. Fang M. Wang X., 2011 ENTPD5, an endoplasmic reticulum UDPase, alleviates ER stress induced by protein overloading in AKT-activated cancer cells. Cold Spring Harb. Symp. Quant. Biol. 76: 217–223. 22169232 10.1101/sqb.2011.76.010876

[bib46] Shi Y. Vattem K. M. Sood R. An J. Liang J., 1998 Identification and characterization of pancreatic eukaryotic initiation factor 2 alpha-subunit kinase, PEK, involved in translational control. Mol. Cell. Biol. 18: 7499–7509. 9819435 10.1128/mcb.18.12.7499 PMC109330

[bib47] Solaini G. Baracca A. Lenaz G. Sgarbi G., 2010 Hypoxia and mitochondrial oxidative metabolism. Biochim. Biophys. Acta 1797: 1171–1177. 20153717 10.1016/j.bbabio.2010.02.011

[bib48] Sriburi R., 2004 XBP1: a link between the unfolded protein response, lipid biosynthesis, and biogenesis of the endoplasmic reticulum. J. Cell Biol. 167: 35–41. 15466483 10.1083/jcb.200406136 PMC2172532

[bib49] Sriburi R. Bommiasamy H. Buldak G. L. Robbins G. R. Frank M., 2006 Coordinate regulation of phospholipid biosynthesis and secretory pathway gene expression in XBP-1(S)-induced endoplasmic reticulum biogenesis. J. Biol. Chem. 282: 7024–7034. 17213183 10.1074/jbc.M609490200

[bib50] Swiech K. Silva C. S. D. Arantes M. K. dos Santos A. S. Astray R. M., 2008 Characterization of growth and metabolism of *Drosophila melanogaster* cells transfected with the rabies-virus glycoprotein gene. Biotechnol. Appl. Biochem. 49: 41. 17570830 10.1042/BA20060148

[bib51] Tavender T. J. Bulleid N. J., 2010 Peroxiredoxin IV protects cells from oxidative stress by removing H_2_O_2_ produced during disulphide formation. J. Cell Sci. 123: 2672–2679. 20627953 10.1242/jcs.067843 PMC2908052

[bib52] Tennessen J. M. Baker K. D. Lam G. Evans J. Thummel C. S., 2011 The *Drosophila* estrogen-related receptor directs a metabolic switch that supports developmental growth. Cell Metab. 13: 139–148. 21284981 10.1016/j.cmet.2011.01.005 PMC3072597

[bib53] Travers K. J. Patil C. K. Wodicka L. Lockhart D. J. Weissman J. S., 2000 Functional and genomic analyses reveal an essential coordination between the unfolded protein response and ER-associated degradation. Cell 101: 249–258. 10847680 10.1016/s0092-8674(00)80835-1

[bib54] Walter P. Ron D., 2011 The unfolded protein response: from stress pathway to homeostatic regulation. Science 334: 1081–1086. 22116877 10.1126/science.1209038

[bib55] Wang M. Kaufman R. J., 2014 The impact of the endoplasmic reticulum protein-folding environment on cancer development. Nat. Rev. Cancer 14: 581–597. 25145482 10.1038/nrc3800

[bib56] Wang S. Kaufman R. J., 2012 The impact of the unfolded protein response on human disease. J. Cell Biol. 197: 857–867. 22733998 10.1083/jcb.201110131 PMC3384412

[bib57] Wang Y. Shen J. Arenzana N. Tirasophon W. Kaufman R. J., 2000 Activation of ATF6 and an ATF6 DNA binding site by the endoplasmic reticulum stress response. J. Biol. Chem. 275: 27013–27020. 10856300 10.1074/jbc.M003322200

[bib58] Wang Z. V. Deng Y. Gao N. Pedrozo Z. Li D. L., 2014 Spliced X-box binding protein 1 couples the unfolded protein response to hexosamine biosynthetic pathway. Cell 156: 1179–1192. 24630721 10.1016/j.cell.2014.01.014 PMC3959665

[bib59] Win S. Than T. A. Fernandez-Checa J. C. Kaplowitz N., 2013 JNK interaction with Sab mediates ER stress induced inhibition of mitochondrial respiration and cell death. Cell Death Dis. 5: e989. 24407242 10.1038/cddis.2013.522 PMC4040675

[bib60] Wu M. Neilson A. Swift A. L. Moran R. Tamagnine J., 2007 Multiparameter metabolic analysis reveals a close link between attenuated mitochondrial bioenergetic function and enhanced glycolysis dependency in human tumor cells. Am. J. Physiol. Cell Physiol. 292: C125–C136. 16971499 10.1152/ajpcell.00247.2006

[bib61] Xi H. Kurtoglu M. Liu H. Wangpaichitr M. You M., 2011 2-Deoxy-D-glucose activates autophagy via endoplasmic reticulum stress rather than ATP depletion. Cancer Chemother. Pharmacol. 67: 899–910. 20593179 10.1007/s00280-010-1391-0 PMC3093301

[bib62] Yoshida H. Matsui T. Yamamoto A. Okada T. Mori K., 2001 XBP1 mRNA is induced by ATF6 and spliced by IRE1 in response to ER stress to produce a highly active transcription factor. Cell 107: 881–891. 11779464 10.1016/s0092-8674(01)00611-0

[bib63] Zheng J., 2012 Energy metabolism of cancer: Glycolysis *vs.* oxidative phosphorylation (Review). Oncol. Lett. 4: 1151–1157. 23226794 10.3892/ol.2012.928 PMC3506713

