# Supplementary material for: Drosophila melanogaster Activating Transcription Factor 4 Regulates Glycolysis During Endoplasmic Reticulum Stress
Source: G3 (Bethesda). 2015 Feb 13;5(4):667–75. doi: 10.1534/g3.115.017269 (PMC4390581; doi:10.1534/g3.115.017269)
Supplement: Supporting Information [file supp_5_4_667__index.html]

Drosophila melanogaster Activating Transcription Factor 4 Regulates Glycolysis During Endoplasmic Reticulum Stress — Supporting Information 

# *Drosophila melanogaster* Activating Transcription Factor 4 Regulates Glycolysis During Endoplasmic Reticulum Stress

## Supporting Information for Lee *et al.*, 2015

**Files in this Data Supplement:**

- Table S1 - Carbon metabolism microarray data, *Drosophila* S2 cells. (.xlsx, 14 KB)
